# Screening and Identification of Candidate GUN1-Interacting Proteins in *Arabidopsis thaliana*

**DOI:** 10.3390/ijms222111364

**Published:** 2021-10-21

**Authors:** Linjuan Wang, Xingqi Huang, Kui Li, Shuyuan Song, Yunhe Jing, Shan Lu

**Affiliations:** 1 State Key Laboratory of Pharmaceutical Biotechnology, School of Life Sciences, Nanjing University, Nanjing 210023, China; linjuan4546@foxmail.com (L.W.); hl_david@msn.com (X.H.); likui@novogene.com (K.L.); songshuyuan131201@163.com (S.S.); jinghomels@163.com (Y.J.); 2 Shenzhen Research Institute of Nanjing University, Shenzhen 518000, China

**Keywords:** chloroplast, DJC31, GUN1, HCF145, protein–protein interaction, retrograde signal

## Abstract

Chloroplasts are semi-autonomous organelles governed by the precise coordination between the genomes of their own and the nucleus for functioning correctly in response to developmental and environmental cues. Under stressed conditions, various plastid-to-nucleus retrograde signals are generated to regulate the expression of a large number of nuclear genes for acclimation. Among these retrograde signaling pathways, the chloroplast protein GENOMES UNCOUPLED 1 (GUN1) is the first component identified. However, in addition to integrating aberrant physiological signals when chloroplasts are challenged by stresses such as photooxidative damage or the inhibition of plastid gene expression, GUN1 was also found to regulate other developmental processes such as flowering. Several partner proteins have been found to interact with GUN1 and facilitate its different regulatory functions. In this study, we report 15 possible interacting proteins identified through yeast two-hybrid (Y2H) screening, among which 11 showed positive interactions by pair-wise Y2H assay. Through the bimolecular fluorescence complementation assay in Arabidopsis protoplasts, two candidate proteins with chloroplast localization, DJC31 and HCF145, were confirmed to interact with GUN1 *in planta.* Genes for these GUN1-interacting proteins showed different fluctuations in the WT and *gun1* mutant under norflurazon and lincomycin treatments. Our results provide novel clues for a better understanding of molecular mechanisms underlying GUN1-mediated regulations.

## 1. Introduction

Chloroplasts are semi-autonomous organelles of photosynthetic eukaryotes. More than 95% of chloroplast proteins are indeed encoded by nuclear genes [1]. In addition, to carry out essential metabolic processes such as photosynthesis and the biosynthesis of amino acids, fatty acids, and phytohormones, chloroplasts also function as a sensor to modulate plant responses to environmental cues [2]. For example, under abiotic stresses and pathogen infection, damaged plastids generate the plastid-to-nucleus retrograde signals to regulate the expression of nuclear genes, including photosynthesis-associated nuclear genes (PhANGs) and genes for chaperones, proteases, nucleases, and defense proteins [1,3,4]. Genetic evidence for the retrograde communication was first demonstrated in *Arabidopsis thaliana* by the characterization of the *genomes uncoupled 1* (*gun1*) mutants, which showed partially rescued expression of PhANGs, compared with the wild type, when chloroplasts were photodamaged under treatment by norflurazon (NF) [5]. From then on, a total of six *GUN* genes have been described [6,7]. Further studies have demonstrated GUN1 as a master component that integrates the aberrant status of the plastid redox state, plastid gene expression, and the tetrapyrrole biosynthesis and generates the retrograde signals [6]. Although other retrograde signaling pathways, such as those via β-cyclocitral and methylerythritol cyclodiphosphate (MEcPP) as signal molecules and the SAL1-PAP pathway have also been identified, the retrograde regulation conveyed by the GUN1 is still one of the best-characterized [8,9,10,11,12].

The GUN1 is a chloroplast localized pentatricopeptide repeat protein (PPR) with a C-terminal small MutS-related (SMR) domain [6]. Besides the rescued expression of the PhANGs under stressed conditions, *gun1* seedlings were also found defective in establishing photoautotrophic growth, suggesting an essential role of GUN1 in the transition from heterotrophic to photoautotrophic growth in germinating seedlings [13]. Recent studies also demonstrated the involvement of GUN1 in other regulatory mechanisms. For example, GUN1 modulates protein homeostasis by controlling the accumulations of plastid ribosomal protein S1 (PRPS1), plays a direct role in RNA editing through its interaction with MORF2, and regulates chloroplast protein import through its interaction with the import-related chaperone cpHSC70-1 [14,15,16]. Moreover, the overexpression of *GUN1* also leads to early flowering phenotype, suggesting a function of GUN1 beyond chloroplast biogenesis and development [17].

GUN1 has been demonstrated to interact with several partner proteins. In addition to PRPS1, MORF2, and cpHSC70-1 mentioned above, GUN1 was also demonstrated to interact with enzymes for tetrapyrrole biosynthesis (such as ChlD) and a cytosolic protein GIP1 [15,16,18]. Although the molecular mechanisms underlying a plethora of GUN1-mediated regulations remain elusive, GUN1 was proposed to function, at least partially, through its physical interaction with different protein partners [14,19,20].

Recently, we performed a yeast two-hybrid (Y2H) screening for possible interacting partners of GUN1. A total of 16 proteins were identified, among which cytosolic GIP1 was found to be recruited to chloroplasts under NF treatment [18]. Here, we report the confirmation of the interactions between GUN1 and two chloroplast proteins, DJC31 and HCF145, by Y2H and biomolecular fluorescence complementation (BiFC) assays.

## 2. Results and Discussion

### 2.1. Y2H Screening

To search for novel interacting proteins of GUN1, we firstly performed a Y2H screening. A truncated peptide of GUN1 with its putative chloroplast transit peptide (cTP) removed (GUN1^ΔcTP^) was used as a bait to screen the Y2H library prepared from one-week-old *Arabidopsis thaliana* seedlings [18]. Positive clones harboring coding regions for 16 candidate interacting proteins, including GIP1, which we recently characterized, were identified from the screening [18]. New candidate proteins are listed in Table 1. Different from the GUN1, which is identified as a chloroplast protein, the candidate interacting proteins are either reported or predicted to have varied subcellular localizations, including cytoplasm, plasma membrane, chloroplasts, and the nucleus, and to possess different metabolic or regulatory functions (Table 1, Table S1).

### 2.2. Pair-Wise Y2H Assay

**Figure 1:**
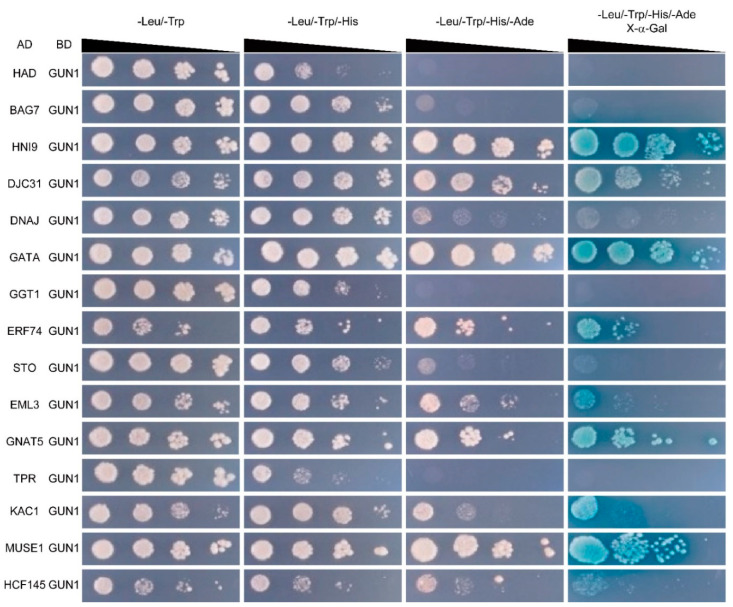
Yeast two-hybrid assay showing the interactions between GUN1 and its candidate partners. GUN1 was fused with the GAL4 DNA binding domain (BD), and each of the candidate proteins was fused with the GAL4 activation domain (AD). Ten-fold serial dilutions of yeast cells expressing different protein pairs as indicated were spotted on non-selective double drop-out (DDO, SD/-Leu/-Trp) and selective triple (TDO, SD/-Leu/-Trp/-His) and quadruple (QDO, SD/-Leu/-Trp/-His/-Ade) drop-out media. To the QDO medium, X-α-Gal was added parallel to indicate the activation of α-galactosidase by the protein–protein interactions.

To validate the result of our Y2H screening, we tested the interaction between GUN1 and each of its candidate partners by pair-wise Y2H assay. The full-length ORF of *GUN1* was cloned into pGBKT7, and the full-length ORFs of genes encoding the candidate proteins were individually cloned into pGADT7. Yeast cells co-expressing GUN1 and each of the 15 partners were able to grow on the selective triple drop-out (TDO) medium, indicating their positive interactions (Figure 1, Figure S1). Among the 15 candidates, 11 proteins might have stronger and more stable interactions with GUN1, as the yeast cells co-expressing each of these proteins with GUN1 were able to grow on a more selective quadruple drop-out (QDO) medium and also showed blue colonies when X-α-Gal was supplemented to the medium (Figure 1, Figure S1).

### 2.3. BiFC Assay

**Figure 2:**
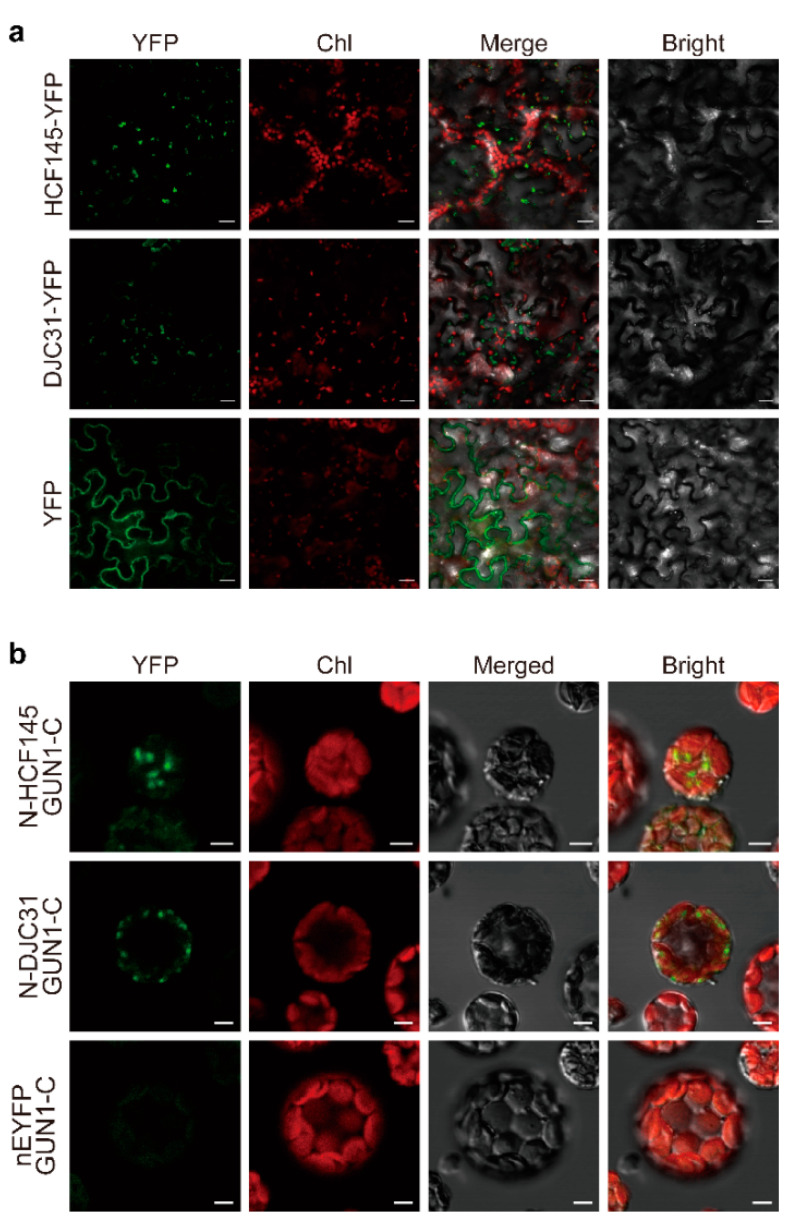
(**a**) Confocal observation showing the chloroplast localization of DJC31 and HCF145. Both proteins were fused upstream of YFP and transiently expressed in tobacco leaves by infiltration. Representative images were collected under the YFP, chlorophyll, bright field channels, and merged (Scale bars, 20 μm), (**b**) BiFC assay demonstrating the interactions between GUN1 and its two interacting proteins. Arabidopsis protoplasts were transfected to express GUN1 and each of its candidate interacting proteins fused with the C- (cEYFP) and N- (nEYFP) halves of EYFP, respectively, as indicated. nEYFP expressed from the empty pSAT1A-nEYFP-N1 vector served as a negative control. Representative images were collected under the YFP, chlorophyll, bright field channels and merged (Scale bars, 5 μm).

To further confirm the identified interactions, we selected two proteins, DJC31 and HCF145, both of which were reported to have chloroplast localization [21,22], and tested their *in planta* interactions with GUN1 by BiFC assays. Because there is a recent report that DJC31 attached to the cytosolic side of the endoplasmic reticulum membrane [23], we checked the subcellular localization of both proteins prior to our analysis. When the YFP fusion proteins of DJC31 and HCF145 were constitutively expressed in the transfected tobacco leaves, their fluorescent signals merged nicely with the chlorophyll autofluorescence from chloroplasts, demonstrating the localization of both proteins at chloroplasts (Figure 2a). This observation did not exclude the presence of these two proteins elsewhere but rationalized their interaction with GUN1 in chloroplasts. We then transformed protoplasts prepared from Arabidopsis mesophyll cells to express GUN1 and its individual candidate partner, fused with cEYFP and nEYFP, respectively, and observed under a confocal microscope. The fluorescent signal of the reconstituted EYFP was observed in the protoplasts co-expressing both fusion protein pairs, indicating their *in planta* interactions (Figure 2b). Moreover, the fluorescent signals of the reconstituted EYFP also merged with chlorophyll autofluorescence (Figure 2b), supporting the view of chloroplasts as the site for their interactions (Figure 2b). As expected, no signal was detectable in the negative controls using an empty vector with GUN1-cEYFP (Figure 2b).

### 2.4. Expression of DJC31 and HCF145 in Response to NF and Lincomycin Treatments

**Figure 3:**
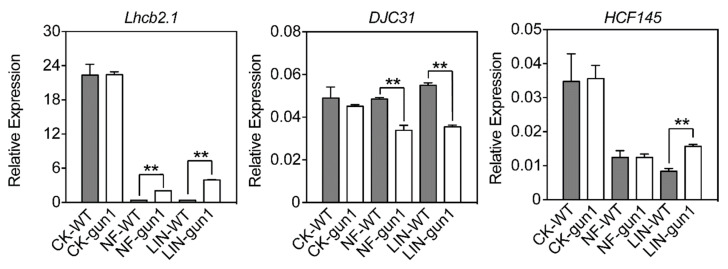
Expression of genes for DJC31 and HCF145 in response to norflurazon and lincomycin treatments. *Arabidopsis thaliana* Col-0 wild-type (WT) and *gun1* mutant seedlings germinated on 1/2 MS plates supplemented with norflurazon (NF, 5 μM) or lincomycin (LIN, 220 μg mL^−1^) under growth condition for 7 d were analyzed. Transcript abundance was determined by qRT-PCR with *Actin2* as a reference. Data represent means ± SD (*n* = 3). Asterisks indicate significant differences between the WT and *gun1* mutant under the same treatment (** *p* < 0.01, Student’s *t* test).

A classic response of the *gun1* mutant to the treatment by norflurazon (inducing photooxidative damage) or lincomycin (inhibiting plastid gene expression) is the rescued expression of PhANGs such as *Lhcb*, compared with the WT seedlings. To assess whether GUN1 or the retrograde signaling pathway regulated genes for DJC31 and HCF145, we treated WT and *gun1* seedlings with NF and lincomycin and quantified the transcript abundance of these genes (Figure S2). Under both treatments, *Lhcb2.1* showed rescued expression levels in the *gun1* mutant, significantly higher than their corresponding WT levels (Figure 3). Different from *Lhcb2.1*, the transcript abundance of *DJC31* was significantly lower in *gun1* than its WT levels under the two treatments (Figure 3). However, *HCF145* has a rescued expression in *gun1* only under lincomycin treatment (Figure 3), suggesting crosstalk in the regulation of plastid gene expression by GUN1 and HCF145 [16,22,24].

Taken together, we identified 15 candidate interacting partners of GUN1. Interestingly, other proteins without chloroplast localization also showed interactions with GUN1 in our Y2H screening. We cannot rule out the possibility of false-positive interactions, which is a common problem in Y2H assays. However, there are also alternative possibilities. First, those candidate proteins might have multiple subcellular localizations. There have been a large number of studies demonstrating proteins with dual localization. For example, the DnaJ-like zinc-finger protein ORANGE was initially identified as a chloroplast protein in leaves and fruits but recently found its nuclear localization in cotyledons of germinating seedlings [25,26,27]. Second, for some candidate proteins, their subcellular localizations were only predicted by software and still need further experimental verification.

By pair-wise Y2H assay and BiFC analysis in this study, we confirmed *in planta* protein–protein interactions between GUN1 and two chloroplast proteins (HCF145 and DJC31) among the 15 candidates. We also demonstrated different transcriptional responses of *DJC31* and *HCF145* in response to NF and lincomycin, the two classical treatments used in the GUN1-related retrograde signaling pathway. Our results suggested the possible involvement of HCF145 and DJC31 in the retrograde regulation mediated by GUN1. Further spatiotemporal characterization of the protein–protein interactions between GUN1 and its interacting partners may provide helpful clues for better understanding the molecular functions of GUN1.

## 3. Methods

### 3.1. Plant Materials and Treatments

Seeds of the *Arabidopsis thaliana* Columbia-0 wild-type (Col-0 WT, CS70000) and the *gun1* mutant (CS833142) were purchased from the Arabidopsis Biological Resource Center (ABRC, Ohio State University, Columbus, OH, USA). Seeds were surface sterilized and stratified at 4 °C for 3 d and then allowed to germinate on 1/2 Murashige and Skoog (MS) medium under 100 μmol photons m^−2^ s^−1^ irradiance with a 16 h/8 h light/dark regime. Although it has been reported that the *gun1* seedlings have altered sensitivity to sucrose in the growth medium, their *gun1* phenotype per se was not affected [28,29]. Therefore, sucrose at 2% was added to the growth medium for helping the growth of germinating seedlings under inhibitor treatments. Tobacco (*Nicotiana benthamiana*) plants were grown from seeds as described previously by Sparkes et al. [30].

To treat *Arabidopsis thaliana* seedlings, norflurazon (NF) at a final concentration of 5 μM or lincomycin at 220 μg mL^−1^ was supplied to the 1/2 MS medium for plant germination [6]. Seven-day-old seedlings were used for gene expression analysis.

### 3.2. Molecular Operation and Gene Expression Quantification

The total RNA was isolated using the RNAiso Plus Reagent (TaKaRa, Shiga, Japan) and reverse transcribed using a HiScript III RT SuperMix for qPCR (+gDNA wiper) kit (Vazyme, Nanjing, China) according to the manufacturers’ instructions. Transcript abundance of each gene was determined by a quantitative real-time PCR (qPCR) in a Thermal Cycler Dice Real-Time System TP800 (TaKaRa) using a ChamQ SYBR qPCR Master Mix (without ROX) (Vazyme) following the manufacturers’ instructions and calculated according to the comparative *C*_T_ method [31]. *Actin2* (*At3g18780*) was quantified as a reference. All primers used in this study are listed in Supplementary Table S2. For each sample, at least three biological replicates were analyzed, and each experiment was repeated three times.

Full-length open reading frames (ORFs) of the genes encoding *GUN1* and its candidate interacting proteins were amplified from the first-strand cDNA pool prepared from the Col-0 WT seedlings as described above. Primers for amplification are listed in Supplemental Table S2. For the *GUN1*, the amplicon was then used as a template for amplifying its truncated version with the 5′-fragment for the putative N-terminal chloroplast transit peptide (cTP, Met^1^-Ser^41^) removed (*GUN1^ΔcTP^*) [18].

### 3.3. Y2H Assays

The Y2H screening was performed by Hybrigenics Services (Paris, France) using a cDNA library prepared from 1-week-old *A. thaliana* seedlings, as previously reported [18].

For the pair-wise Y2H assay to confirm the protein–protein interactions identified from the Y2H screening, full-length ORF of *GUN1* was fused downstream of the DNA-binding domain (BD) of pGBK-T7 as a bait, and the full-length ORF of each of the genes for GUN1-interacting proteins was fused downstream of the activation domain (AD) of pGAD-T7 as a prey. Yeast (*Saccharomyces cerevisiae* strain AH109) cells transformed with both bait and prey constructs were screened on non-selective double drop-out (DDO, SD/-Leu/-Trp), selective triple drop-out (TDO, SD/-Leu/-Trp/-His), and quadruple drop-out (QDO, SD/-Leu/-Trp/-His/-Ade) media. To QDO medium, 5-bromo-4-chloro-3-indolyl-β-d-galactopyranoside (X-α-Gal) was added in parallel to detect the activation of an α-galactosidase by the protein–protein interactions. Empty vectors were used as negative controls. Transformed yeast cells were grown for 5 d at 30 °C before representative images were taken.

### 3.4. BiFC Assay

Bioinformatic information of the subcellular localization of the GUN1-interacting proteins was retrieved from SUBA4 (https://suba.live) [32].

For subcellular localization analysis, full-length ORFs of *DJC31* and *HCF145* were individually cloned into the NcoI site of pCNHP-EYFP, which we constructed based on the pCAMBIA1300, and harbored sequentially the enhanced Cauliflower mosaic virus (CaMV) *35S* promoter, synthetic 5′ and 3′ untranslated regions of Cowpea mosaic virus RNA2 flanking the coding region fused in frame to the 5′-end of the gene for either enhanced yellow fluorescent protein (EYFP), and the Heat Shock Protein (HSP) terminator from *A. thaliana* [33], to generate 35S:DJC3-EYFP and 35S:HCF145-EYFP, respectively. The transformation and cultivation of *Agrobacterium tumefaciens* strain GV3101 and the infiltration of *Nicotiana benthamiana* leaves were performed as described [33,34]. Signals of the constitutively expressed fluorescent fusion proteins were observed at 3-d after infiltration using a FLUOVIEW FV1000 Laser Confocal Microscopy System (Olympus, Tokyo, Japan).

For detecting *in planta* protein–protein interactions, the full-length ORF of each of the candidate genes for GUN1-interacting proteins was cloned into pSAT1A-nEYFP-N1 (ABRC), whereas that of the GUN1 was cloned into pSAT1A-cEYFP-N1 (ABRC). Primers used for generating corresponding constructs are listed in Supplemental Table S2.

For transient expression of a pair of fusion proteins for both GUN1 and its interacting protein in protoplasts, we isolated protoplasts from *A. thaliana* leaves and performed the PEG-mediated transfection as described with a few minor modifications [35]. In brief, green tissues of *A. thaliana* WT seedlings were cut into approximately 0.5–1 mm strips, which were immediately transferred into the freshly prepared enzyme solution (1% cellulose R10, 0.3% macerozyme R10, 0.4 M mannitol, 0.3 M MES, pH 5.7, 0.3 M KCl, 0.75 mM β-mercaptoethanol, 1.5% BSA) and vacuum infiltrated for 2 min at room temperature. Leaf stripes were enzymatically digested at 22 °C for 3 h in the dark with gentle shaking. After digestion, an equal volume of W5 solution (154 mM NaCl, 125 mM CaCl_2_, 5 mM KCl, 5 mM D-glucose, 2 mM MES, pH 5.7) was added, and protoplasts were released and isolated by filtrating through 100 μm nylon meshes. After being centrifuged at 100× *g* for 2 min, pelleted protoplasts were resuspended with 5 mL W5 solution, recovered on ice for 40 min, re-pelleted by centrifugation at 100× *g* for 2 min, and then suspended in MaMg solution (15 mM MgCl_2_, 0.4 M mannitol, 0.1% MES, pH 5.7) at a concentration of 2 × 10^5^ protoplasts/mL counted under a hematocytometer. PEG-mediated transfection was performed as described [35]. Plasmid DNA (15 μL, 2 μg/μL) was mixed with 100 μL protoplasts, and then 110 μL freshly prepared PEG solution (50% PEG4000, 0.8 M mannitol, 1 M CaCl_2_) by gently tapping. The mixture was incubated at room temperature for 15 min before mixing with 440 μL W5 solution. Protoplasts were collected by centrifugation at 100× *g* for 2 min and resuspended with 250 μL W5 solution. Finally, protoplasts were cultured in 24-well plates at 22 °C for 16 h in the dark as previously described [36].

For detecting EYFP, the excitation wavelength was 488 nm, and the emission filter was 500–530 nm. The chlorophyll autofluorescence was monitored using 543 nm excitation wavelength and 650–755 nm detection window. All figures show representative images from at least five independent experiments.

### 3.5. Statistical Analysis

GraphPad Prism 7 (GraphPad Software, San Diego, CA, USA) was used for statistical analysis. To determine statistical significance, we employed Student’s *t* test. Differences were considered significant at *p* < 0.01 levels.

## Figures and Tables

**Table 1:** GUN1-interacting proteins identified by yeast two-hybrid screening.

Name	AGI No.	Predicted Localization
BAG7	AT5G62390	endoplasmic reticulum
DJC31 *	AT5G12430	chloroplast
DNAJ *	AT2G25560	cytoplasm
EML3 *	AT5G13020	nucleus
ERF74 *	AT1G53910	nucleus
GATA *	AT1G28400	extracellular
GGT1	AT1G23310	peroxisome
GNAT5 *	AT1G24040	chloroplast
HAD	AT5G36790	chloroplast
HCF145 *	AT5G08720	chloroplast
HNI9 *	AT1G32130	nucleus
KAC1 *	AT5G10470	cytoplasm
MUSE1 *	AT3G58030	nucleus
STO *	AT1G06040	nucleus
TPR	AT5G28740	nucleus

* Interaction with GUN1 was further confirmed by pair-wise Y2H assay.

## Supplementary Materials

The following are available online at https://www.mdpi.com/article/10.3390/ijms222111364/s1.
