# Supplementary material for: Screening and Identification of Candidate GUN1-Interacting Proteins in Arabidopsis thaliana
Source: Int J Mol Sci. 2021 Oct 21;22(21):11364. doi: 10.3390/ijms222111364 (PMC8583188; doi:10.3390/ijms222111364)
Supplement: Supplementary file 1 [file ijms-22-11364-s001.zip › Supplemental figures.pdf]

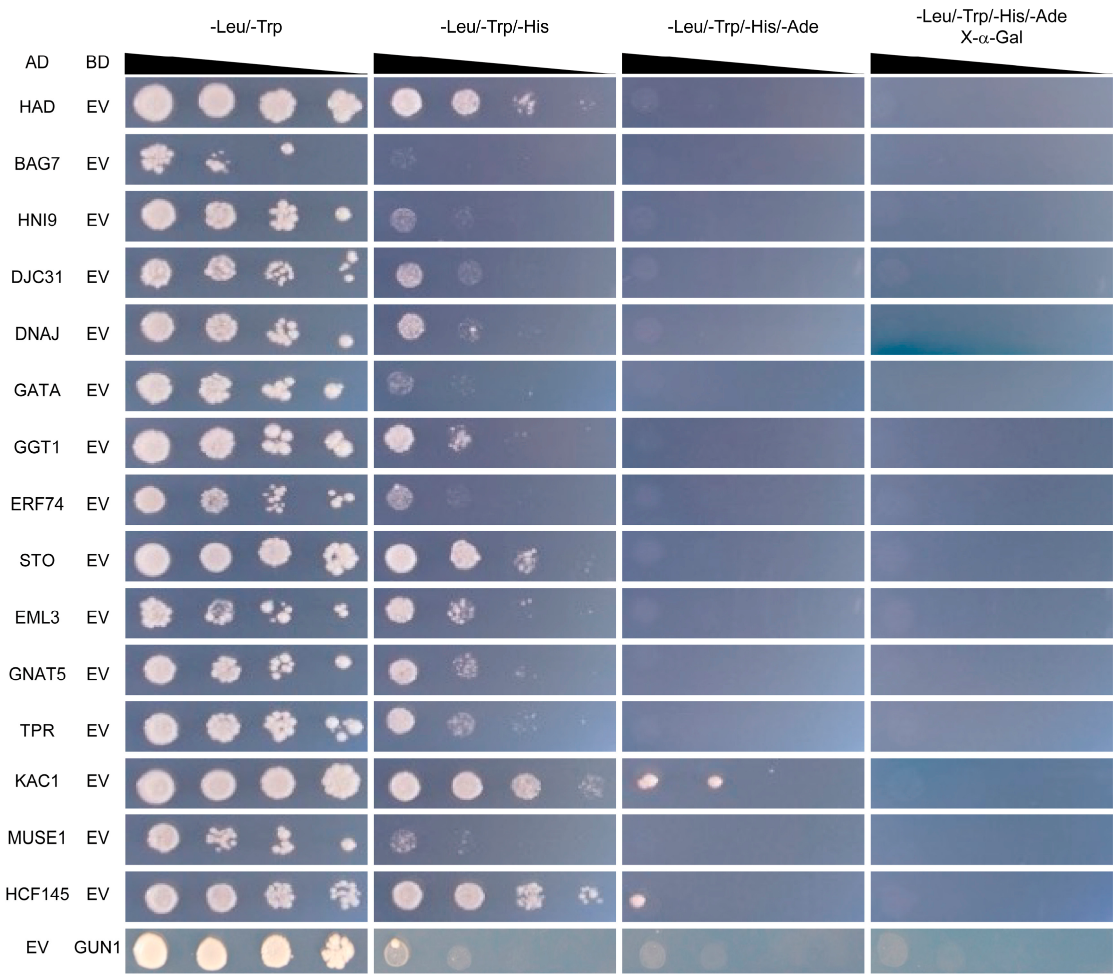

Figure S1. Negative controls of the pair-wise yeast two-hybrid assay.

GUN1 was fused with the GAL4 DNA binding domain (BD), and each of the candidate proteins was fused with the GAL4 activation domain (AD). Empty vectors (EVs) served as negative controls. Ten-fold serial dilutions of yeast cells expressing different protein pairs as indicated were spotted on non-selective double drop-out (DDO, SD/-Leu/-Trp) and selective triple (TDO, SD/-Leu/-Trp/-His) and quadruple (QDO, SD/-Leu/-Trp/-His/-Ade) drop-out media. To the QDO medium, X-α-Gal was added in parallel to indicate the activation of α-galactosidase by the protein-protein interactions.

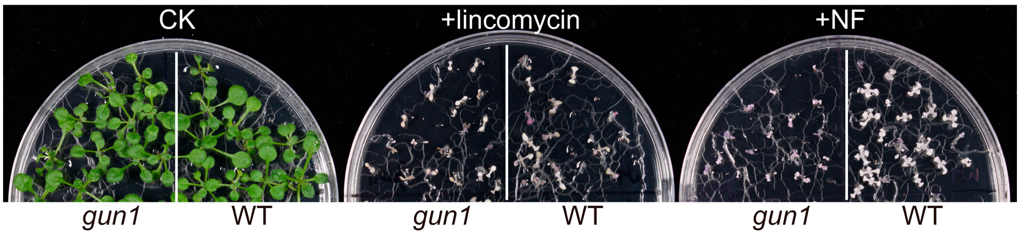

Figure S2. Phenotype of WT and *gun1* seedlings germinating on growth medium supplemented with norflurazon or lincomycin.

Seeds of both *Arabidopsis thaliana* WT and *gun1* mutant were stratified stratified at 4 °C for 3 d in the dark, and then germinated on 1/2 MS medium (containing 2% sucrose and 0.8% agar) supplemented with norflurazon (NF, 5 μM) or lincomycin (220 μg mL^{-1}). Growth medium without inhibitor supplement was used as a control (CK). One-week-old seedlings were photographed.
