# Supplementary material for: Screening and Identification of Candidate GUN1-Interacting Proteins in Arabidopsis thaliana
Source: Int J Mol Sci. 2021 Oct 21;22(21):11364. doi: 10.3390/ijms222111364 (PMC8583188; doi:10.3390/ijms222111364)
Supplement: Supplementary file 1 [file ijms-22-11364-s001.zip › Supplemental Table S1.pdf]

Supplemental Information

Table S1. Primers used in this study.

Primer name	Primer sequence
For gene cloning	
GUN1-HF	ATGGCGTCAACGCCGCCTCACTGGGTCAC
GUN1-ER	CTACAAAAGAAGAGGCTGTAAAGCAAACGACGTC
STO-HF	ATGAAGATACAGTGTGATGTGTGTGAG
STO-ER	TTAGCCAAGATCAGGGACAATGAAG
GGT1-HF	ATGGCTCTCAAGGCATTAGACTACG
GGT1-ER	TCACATTTTCGAATAACCAAAGTTATTATC
NAT-HF	ATGGCGGCTTTAAGCATCTCAC
NAT-ER	TTACACATTTGCAGAGGAGGTCAGC
GATA-HF	ATGGCTTTCTCCACTAGAGGCT
GATA-ER	TCAAGGCTTGAACTGATTAGTGCC
HNI9-HF	ATGGGTTTCGAGGATGATCCGTAC
HNI9-ER	CTAGAGGTACTTGATCATACCCCGAC
ERF74-HF	ATGTGTGGAGGAGCTATAATATCCG
ERF74-ER	TCAGAAGACTCCTCCAATCATGGAATG
DNAJ-HF	ATGGAGTTTAATAAAGAAGAAGCTACAAG
DNAJ-ER	TTACCGATAATCATCGATAGCCTGAAG
MUSE1-HF	ATGGGTGAGGAGTTAGCTGACAC
MUSE1-ER	TCAAGTAAATCTCCTTCTCCTAGGC
For Y2H assay	
GUN1-BD-F	GCATATGGCCATGGAGGCCGAATTCATGGCGTCAACGCCG
GUN1-BD-R	CAGGTCGACGGATCCCCGGGAATTCCTACAAAAGAAGAGGCTGTAAAGCAAACG
AD-STO-F	CATATGGCCATGGAGGCCAGTGAATTCATGAAGATACAGTGTGATGTGTGTGAG
AD-STO-R	GTATCGATGCCCACCCGGGTGGAATTCTTAGCCAAGATCAGGGACAATGAAG
AD-GGT1-F	GCTCATATGGCCATGGAGGCCAGTGAATTCATGGCTCTCAAGGCATTAGACTACG
AD-GGT1-R	CGTATCGATGCCCACCCGGGTGGAATTCTCACATTTTCGAATAACCAAAGTTATTATC
AD-NAT-F	GCTCATATGGCCATGGAGGCCAGTGAATTCATGGCGGCTTTAAGCATCTCAC
AD-NAT-R	GTATCGATGCCCACCCGGGTGGAATTCTTACACATTTGCAGAGGAGGTCAGC
AD-GATA-F	GCTCATATGGCCATGGAGGCCAGTGAATTCATGGCTTTCTCCACTAGAGGCT
AD-GATA-R	GTATCGATGCCCACCCGGGTGGAATTCTCAAGGCTTGAACTGATTAGTGCC
AD-HNI9-F	GCTCATATGGCCATGGAGGCCAGTGAATTCATGGGTTTCGAGGATGATCCGT
AD-HNI9-R	GTATCGATGCCCACCCGGGTGGAATTCCTAGAGGTACTTGATCATACCCCGAC
AD-ERF74-F	GCTCATATGGCCATGGAGGCCAGTGAATTCATGTGTGGAGGAGCTATAATATCCGA
AD-ERF74-R	GTATCGATGCCCACCCGGGTGGAATTCTCAGAAGACTCCTCCAATCATGGAATG
AD-DNAJ-F	CATATGGCCATGGAGGCCAGTGAATTCATGGAGTTTAATAAAGAAGAAGCTACAAG
AD-DNAJ-R	GTATCGATGCCCACCCGGGTGGAATTCTTACCGATAATCATCGATAGCCTGAAG
AD-MUSE1-F	GCTCATATGGCCATGGAGGCCAGTGAATTCATGGGTGAGGAGTTAGCTGACAC
AD-MUSE1-R	CCGTATCGATGCCCACCCGGGTGGAATTCTCAAGTAAATCTCCTTCTCCTAGGC
AD-HCF145-F	GCTCATATGGCCATGGAGGCCAGTGAATTCATGTCAGTGAGCAAGTTTCCACATC
AD-HCF145-R	CCCGTATCGATGCCCACCCGGGTGGAATTCTCAATATTGAACCCAATTGATATCAAG
AD-KAC1-F	GCTCATATGGCCATGGAGGCCAGTGAATTCATGGCCGATCAGAGAAGTAAAACCA
AD-KAC1-R	GTATCGATGCCCACCCGGGTGGAATTCTTACTCCAGTTCACTAACAAGGTCC
AD-DJC31-F	GCTCATATGGCCATGGAGGCCAGTGAATTCATGAGCAAGTTCGGCGAATTGAA

AD-DJC31-R	CGTATCGATGCCCACCCGGGTGGAATTCTTACGGGTATCTGTTTGATCGGTTTGG
AD-EML3-F	CTCATATGGCCATGGAGGCCAGTGAATTCATGGATTACCGACCTTCTGATAGTAGT
AD-EML3-R	CCCGTATCGATGCCCACCCGGGTGGAATTCTCAAATATTACCGCTTTCTCCATCTG
AD-TPR-F	GCTCATATGGCCATGGAGGCCAGTGAATTCATGGCGATTTCCAAAGATCTCTATCC
AD-TPR-R	CCCGTATCGATGCCCACCCGGGTGGAATTCTCACTGATTAAGCTTCTGTCTCTTG
AD-HAD-F	GCTCATATGGCCATGGAGGCCAGTGAATTCATGCTGAGTAGATCAGTTGCTTCTG
AD-HAD-R	GTATCGATGCCCACCCGGGTGGAATTCTTATACAGTTGCGGCTTTCGGAGAC
AD-BAG7-F	GCTCATATGGCCATGGAGGCCAGTGAATTCATGACTTTGTTCCATAGACTCGATCTC
AD-BAG7-R	CCCGTATCGATGCCCACCCGGGTGGAATTCTCATTCTTCTTCCGTTTCAAGCATC
For BiFC assay	
BIFC-N-HIN9-F	CTGTATATTCTGCCCAAATTCGCGCCATGGATGGGTTTCGAGGATGATCCG
BIFC-N-HIN9-R	CAGCTCCTCGCCCTTGCTCACCATCCATGGGAGGTACTTGATCATACCCCGA
BIFC-N-GATA-F	CTGTATATTCTGCCCAAATTCGCGCCATGGATGGCTTTCTCCACTAGAGGCT
BIFC-N-GATA-R	CTCCTCGCCCTTGCTCACCATCCATGGAGGCTTGAACTGATTAGTGCCTTG
BIFC-N-MUSE1-F	CTGTATATTCTGCCCAAATTCGCGCCATGGATGGGTGAGGAGTTAGCTGACAC
BIFC-N-MUSE1-R	CAGCTCCTCGCCCTTGCTCACCATCCATGGAGTAAATCTCCTTCTCCTAGG
BiFc-N-DJC31-F	CTGTATATTCTGCCCAAATTCGCGCCATGGATGAGCAAGTTCGGCGAATTGAA
BiFc-N-DJC31-R	GCTCCTCGCCCTTGCTCACCATCCATGGCGGGTATCTGTTTGATCGGT
BiFc-N-EML3-F	CTGTATATTCTGCCCAAATTCGCGCCATGGATGGATTACCGACCTTCTGATAG
BiFc-N-EML3-R	CTCGCCCTTGCTCACCATCCATGGAATATTACCGCTTTCTCCATCTGATATTTC
BiFc-N-HCF145-F	GTATATTCTGCCCAAATTCGCGCCATGGATGTCAGTGAGCAAGTTTCCACAT
BiFc-N-HCF145-R	CCTCGCCCTTGCTCACCATCCATGGATATTGAACCCAATTGATATCAAGATC
BiFC-C-GUN1-F	CTGTATATTCTGCCCAAATTCGCGCATGGCGTCAACGCCG
BIFC-C-GUN1-R	TCGGCGAGCTGCACGCTGCCCCAAAAGAAGAGGCTGTAAAGCAAACG
pSAT1A-C-GUN1-F	ATAGAGATCTCGAGCTCAAGCTTCGAATTCATGGCGTCAACGCCG
pSAT1A-C-GUN1-R	CCGCGGTACCGTCGACTGCAGAATTCCAAAAGAAGAGGCTGTAAAGCAAACGAC
pSAT4A-N-HNI9-F	ATAGAGATCTCGAGCTCAAGCTTCGAATTCATGGGTTTCGAGGATGATCCGT
pSAT4A-N-HNI9-R	CCCGCGGTACCGTCGACTGCAGAATTCGAGGTACTTGATCATACCCCGACC
pSAT4A-N-GATA-F	ATAGAGATCTCGAGCTCAAGCTTCGAATTCATGGCTTTCTCCACTAGAGGCT
pSAT4A-N-GATA-R	CCCGCGGTACCGTCGACTGCAGAATTCAGGCTTGAACTGATTAGTGCCTTG
pSAT4A-N-MUSE1-F	ATAGAGATCTCGAGCTCAAGCTTCGAATTCATGGGTGAGGAGTTAGCTGACAC
pSAT4A-N-MUSE1-R	CCCGCGGTACCGTCGACTGCAGAATTCAGTAAATCTCCTTCTCCTAGGCGG
PSAT4A-N-HCF145-F	ATAGAGATCTCGAGCTCAAGCTTCGAATTCATGTCAGTGAGCAAGTTTCCACATC
PSAT4A-N-HCF145-R	CCCGCGGTACCGTCGACTGCAGAATTCATATTGAACCCAATTGATATCAAGATC
PSAT4A-N-DJC31-F	ATAGAGATCTCGAGCTCAAGCTTCGAATTCATGAGCAAGTTCGGCGAATTGAA
PSAT4A-N-DJC31-R	CGCGGTACCGTCGACTGCAGAATTCCGGGTATCTGTTTGATCGG
PSAT4A-N-EML3-F	ATAGAGATCTCGAGCTCAAGCTTCGAATTCATGGATTACCGACCTTCTGATAGT
PSAT4A-N-EML3-R	CGCGGTACCGTCGACTGCAGAATTCAATATTACCGCTTTCTCCATCTGATATTTC
pSAT4A-N-ERF74-F	ATAGAGATCTCGAGCTCAAGCTTCGAATTCATGTGTGGAGGAGCTATAATATCCG
pSAT4A-N-ERF74-R	CGCGGTACCGTCGACTGCAGAATTCGAAGACTCCTCCAATCATGGAATGAATTTC
For qRT-PCR	
Actin-F	CCAACATATGCATCCTTCTGGTTCATCCCA
Actin-R	TGGCTGAGGCTGATGATATTCAACCAATCG
Lhcb2.1-F	GTGACCATGCGTCGTACCGTC
Lhcb2.1-R	CTCAGGGAATGTGCATCCGAG
GLK1-F	CCGTATTTACCGACCGTAGCTACGAGA
GLK1-R	TACATCGTGTGATGCGGCGGCAGAG

MUSE1-F	CCCGCCTGAGAGATCTTCTGAAGC
MUSE1-R	CGCTTTCTTATCCAATACACCGTCCCC
DJC31-F	GGTGAGAAGGCAAGTAAAGTCTCAG
DJC31-R	GCCTTGAAGCCTTTCCTTCACTCTC
HNI9-F	GATGTAGATGGAGAGCCAATAGTGGAC
HNI9-R	CATCCACATCCTCATCAATCAATTCCGG
GATA-F	GCGAGAAAACCACAGTAGAAGAATCCG
GATA-R	GTTGAAAGACTCTTCGGTCTCGCTC
HCF145-F	GTGAGCAAGTTTCCACATCTCTCTCATG
HCF145-R	CGAGATCACATCAACCTCACAACGC
ERF74-F	GCTCGAAGAATCGTTCGAATTTCTTCG
ERF74-R	GCCTAATCCCTCGGTACTGATTCTTCC
