# Supplementary material for: Screening and Identification of Candidate GUN1-Interacting Proteins in Arabidopsis thaliana
Source: Int J Mol Sci. 2021 Oct 21;22(21):11364. doi: 10.3390/ijms222111364 (PMC8583188; doi:10.3390/ijms222111364)
Supplement: Supplementary file 1 [file ijms-22-11364-s001.zip › Supplemental Table S2-plain.pdf]

Table S2. Annotation of GUN1-interacting proteins identified in this study.

Name	AGI No.	Annotation	References
BAG7	AT5G62390	A member of Arabidopsis BAG (Bcl-2-associated athanogene) proteins, plant homologs of mammalian regulators of apoptosis. Plant BAG proteins are multi-functional and remarkably similar to their animal counterparts, as they regulate apoptotic-like processes ranging from pathogen attack, to abiotic stress, to plant development. Localized to the ER. Necessary for the proper maintenance of the unfolded protein response during heat and cold tolerance.	(Pan et al. 2016; Zhou et al. 2021; Li et al. 2017; Williams et al. 2010)
DJC31*	AT5G12430	Encodes one of the 36 carboxylate clamp (CC)-tetratricopeptide repeat (TPR) proteins with potential to interact with Hsp90/Hsp70 as co-chaperones.	
DNAJ	AT2G25560	DNAJ heat shock N-terminal domain-containing protein	
EML3*	AT5G13020	Agenet domain containing nucleosome binding protein. Binds H3K36 sites	(Coursey et al. 2018; Milutinovic et al. 2019)
ERF74*	AT1G53910	Encodes a member of the ERF (ethylene response factor) subfamily B-2 of ERF/AP2 transcription factor family (RAP2.12). The protein contains one AP2 domain. There are 5 members in this subfamily including RAP2.2 AND RAP2.12. Involved in oxygen sensing. It plays a key role in controlling root bending in response to hypoxia.	(Giuntoli et al. 2017; White et al. 2018; Schmidt et al. 2018; Valeri et al. 2021; Shukla et al. 2020)
GATA*	AT1G28400	GATA zinc finger protein;	
GGT1	AT1G23310	Identified by cloning the gene that corresponded to a purified protein having glyoxylate aminotransferase activity. Localized to the peroxisome and thought to be involved in photorespiration/ metabolic salvage pathway.	(Dellero et al. 2015; Laxa et al. 2016; Gonzalez-Lopez et al. 2021)
HAD	AT5G36790	Haloacid dehalogenase-like hydrolase (HAD) superfamily protein	
HCF145*	AT5G08720	Encodes PIN2 PROMOTER BINDING PROTEIN 1 (PPP1), an evolutionary conserved plant-specific DNA binding protein that acts on transcription of PIN genes. Also named as HCF145. Mutations in HCF145 have reduced level of the tricistronic psaA-psaB-rps (small-subunit ribosomal protein)14 mRNA which encodes for the major subunits of the photosystem I (PSI). HCF145 binds to the 5'UTR of PSAA via a novel TMR domain. It functions to stabilize the PSAA transcript.	(Manavski et al. 2015)
HNI9*	AT1G32130	The C-terminal portion of this protein has high homology to the C-termini of the IWS1 (Interacts With Spt6) proteins found in yeast and humans. Interacts with transcription factor BES1. Involved in brassinosteroid-regulated gene expression.	(Li et al. 2010)

KAC1	AT5G10470	Kinesin that binds cyclin-dependent kinase CDKA;1 as homodimer or as heterodimer with KCA2. Demarcates the division site in plant cells.	(Vanstraelen et al. 2006)
MUSE1*	AT3G58030	Encodes a RING domain E3 ligase. Has overlapping function with MUSE2 in the negative regulation of defence responses. SIKIC2 (and possibly SIKIC1 and 3) is ubiquination target.	
GNAT5	AT1G24040	Acyl-CoA N-acyltransferases (NAT) superfamily protein;	
STO	AT1G06040	Encodes salt tolerance protein (STO) which confers salt tolerance to yeast cells. Fully complements calcineurin deficient yeast but does not encode a phosphoprotein phosphatase. Sequence has similarities to CONSTANS. STO co-localizes with COP1 and plays a role in light signaling.STO transcript levels are regulated by photoperiod and phtyohormones. STO competes with FLC in the regulation of floral transition genes SOC1 and FT.	(Li et al. 2013)
TPR	AT5G28740	Tetratricopeptide repeat (TPR)-like superfamily protein	

*Interaction with GUN1 was further confirmed by BiFC assay.

References

Coursey T, Milutinovic M, Regedanz E, Brkljacic J, Bisaro DM (2018) Arabidopsis histone reader EMSY-LIKE 1 binds H3K36 and suppresses geminivirus infection. J Virol 92:00219-18. https://doi.org/10.1128/JVI.00219-18

Dellero Y, Lamothe-Sibold M, Jossier M, Hodges M (2015) *Arabidopsis thaliana ggt1* photorespiratory mutants maintain leaf carbon/nitrogen balance by reducing RuBisCO content and plant growth. Plant J 83:1005-1018. https://doi.org/10.1111/tpj.12945

Giuntoli B, Shukla V, Maggiorelli F, Giorgi FM, Lombardi L, Perata P, Licausi F (2017) Age-dependent regulation of ERF-VII transcription factor activity in *Arabidopsis thaliana*. Plant Cell Environ 40:2333-2346. https://doi.org/10.1111/pce.13037

Gonzalez-Lopez MDC, Jijon-Moreno S, Dautt-Castro M, Ovando-Vazquez C, Ziv T, Horwitz BA, Casas-Flores S (2021) Secretome analysis of *Arabidopsis-Trichoderma atroviride* interaction unveils new roles for the plant Glutamate:Glyoxylate Aminotransferase GGAT1 in plant growth induced by the fungus and resistance against *Botrytis cinerea*. Int J Mol Sci 22:6804. https://doi.org/10.3390/ijms22136804

Laxa M, Muller K, Lange N, Doering L, Pruscha JT, Peterhansel C (2016) The 5' UTR intron of Arabidopsis GGT1 aminotransferase enhances promoter activity by recruiting RNA Polymerase II. Plant Physiol 172:313-327. https://doi.org/10.1104/pp.16.00881

Li L, Ye H, Guo H, Yin Y (2010) Arabidopsis IWS1 interacts with transcription factor BES1 and is involved in plant steroid hormone brassinosteroid regulated gene expression. Proc Natl Acad Sci USA 107:3918-3923. https://doi.org/10.1073/pnas.0909198107

Li X, Gao Q, Liang Y, Ma T, Cheng L, Qi D, Liu H, Xu X, Chen S, Liu G (2013) A novel salt-induced gene from sheepgrass, *LcSAIN2*, enhances salt tolerance in transgenic Arabidopsis. Plant Physiol Biochem 64:52-59. https://doi.org/10.1016/j.plaphy.2012.12.014

Li Y, Williams B, Dickman M (2017) Arabidopsis B-cell lymphoma2 (Bcl-2)-associated athanogene 7 (BAG7)-mediated heat tolerance requires translocation, sumoylation and binding to WRKY29. New Phytol 214:695-705. https://doi.org/10.1111/nph.14388

Manavski N, Torabi S, Lezhneva L, Arif MA, Frank W, Meurer J (2015) HIGH CHLOROPHYLL FLUORESCENCE145 binds to and stabilizes the *psaA* 5' UTR via a newly defined repeat motif in Embryophyta. Plant Cell 27:2600-2615. https://doi.org/10.1105/tpc.15.00234

Milutinovic M, Lindsey BE, 3rd, Wijeratne A, Hernandez JM, Grotewold N, Fernandez V, Grotewold E, Brkljacic J (2019) Arabidopsis EMSY-like (EML) histone readers are necessary for post-fertilization seed development, but prevent fertilization-independent seed formation. Plant Sci 285:99-109. https://doi.org/10.1016/j.plantsci.2019.04.007

Pan YJ, Liu L, Lin YC, Zu YG, Li LP, Tang ZH (2016) Ethylene antagonizes salt-induced growth retardation and cell death process via transcriptional controlling of ethylene-, BAG- and senescence-associated genes in Arabidopsis. Front Plant Sci 7:696. https://doi.org/10.3389/fpls.2016.00696

Schmidt RR, Fulda M, Paul MV, Anders M, Plum F, Weits DA, Kosmacz M, Larson TR, Graham IA, Beemster GTS, Licausi F, Geigenberger P, Schippers JH, van Dongen JT (2018) Low-oxygen response is triggered by an ATP-dependent shift in

oleoyl-CoA in Arabidopsis. Proc Natl Acad Sci USA 115:E12101-E12110. https://doi.org/10.1073/pnas.1809429115

Shukla V, Lombardi L, Pencik A, Novak O, Weits DA, Loreti E, Perata P, Giuntoli B, Licausi F (2020) Jasmonate signalling contributes to primary root inhibition upon oxygen deficiency in *Arabidopsis thaliana*. Plants (Basel) 9:1046. https://doi.org/10.3390/plants9081046

Valeri MC, Novi G, Weits DA, Mensuali A, Perata P, Loreti E (2021) *Botrytis cinerea* induces local hypoxia in Arabidopsis leaves. New Phytol 229:173-185. https://doi.org/10.1111/nph.16513

Vanstraelen M, Van Damme D, De Rycke R, Mylle E, Inzé D, Geelen D (2006) Cell cycle-dependent targeting of a kinesin at the plasma membrane demarcates the division site in plant cells. Curr Biol 16:308-314. https://doi.org/10.1016/j.cub.2005.12.035

White MD, Kamps J, East S, Taylor Kearney LJ, Flashman E (2018) The plant cysteine oxidases from *Arabidopsis thaliana* are kinetically tailored to act as oxygen sensors. J Biol Chem 293:11786-11795. https://doi.org/10.1074/jbc.RA118.003496

Williams B, Kabbage M, Britt R, Dickman MB (2010) *AtBAG7*, an *Arabidopsis* Bcl-2-associated athanogene, resides in the endoplasmic reticulum and is involved in the unfolded protein response. Proc Natl Acad Sci USA 107:6088-6093. https://doi.org/10.1073/pnas.0912670107

Zhou Y, Yang K, Cheng M, Cheng Y, Li Y, Ai G, Bai T, Xu R, Duan W, Peng H, Li X, Xia A, Wang Y, Jing M, Dou D, Dickman MB (2021) Double-faced role of Bcl-2-associated athanogene 7 in plant-*Phytophthora* interaction. J Exp Bot 72:5751-5765. https://doi.org/10.1093/jxb/erab252
